# Do patients with high versus low treatment and illness burden have different needs? A mixed-methods study of patients living on dialysis

**DOI:** 10.1371/journal.pone.0260914

**Published:** 2021-12-28

**Authors:** Kasey R. Boehmer, Kathleen H. Pine, Samantha Whitman, Paige Organick, Anjali Thota, Nataly R. Espinoza Suarez, Christina M. LaVecchia, Alexander Lee, Emma Behnken, Bjorg Thorsteinsdottir, Aditya S. Pawar, Annika Beck, Elizabeth C. Lorenz, Robert C. Albright

**Affiliations:** 1 Knoweldge and Evaluation Research (KER) Unit, Mayo Clinic, Rochester, Minnesota, United States of America; 2 College of Health Solutions, Arizona State University, Phoenix, Arizona, United States of America; 3 Human & Social Dimensions of Science & Technology, Arizona State University, Phoenix, Arizona, United States of America; 4 Neumann University, Aston, Pennsylvania, United States of America; 5 Health Services Research, Mayo Clinic, Rochester, Minnesota, United States of America; 6 Community Internal Medicine, Mayo Clinic, Rochester, Minnesota, United States of America; 7 Neprhology and Hypertension, Mayo Clinic, Rochester, Minnesota, United States of America; 8 Beth Israel Deaconess Medical Center, Boston, Massachusetts, United States of America; 9 Bioethics, Mayo Clinic, Rochester, Minnesota, United States of America; Faculty of Health Sciences - Universidade da Beira Interior, PORTUGAL

## Abstract

**Background:**

Approximately 750,000 people in the U.S. live with end-stage kidney disease (ESKD); the majority receive dialysis. Despite the importance of adherence to dialysis, it remains suboptimal, and one contributor may be patients’ insufficient capacity to cope with their treatment and illness burden. However, it is unclear what, if any, differences exist between patients reporting high versus low treatment and illness burden.

**Methods:**

We sought to understand these differences using a mixed methods, explanatory sequential design. We enrolled adult patients receiving dialysis, including in-center hemodialysis, home hemodialysis, and peritoneal dialysis. Descriptive patient characteristics were collected. Participants’ treatment and illness burden was measured using the Illness Intrusiveness Scale (IIS). Participants scoring in the highest quartile were defined as having high burden, and participants scoring in the lowest quartile as having low burden. Participants in both quartiles were invited to participate in interviews and observations.

**Results:**

Quantitatively, participants in the high burden group were significantly younger (mean = 48.4 years vs. 68.6 years respectively, p = <0.001). No other quantitative differences were observed. Qualitatively, we found differences in patient self-management practices, such as the high burden group having difficulty establishing a new rhythm of life to cope with dialysis, greater disruption in social roles and self-perception, fewer appraisal focused coping strategies, more difficulty maintaining social networks, and more negatively portrayed experiences early in their dialysis journey.

**Conclusions and relevance:**

Patients on dialysis reporting the greatest illness and treatment burden have difficulties that their low-burden counterparts do not report, which may be amenable to intervention.

## Introduction

Three quarters of a million people in the United States live with end-stage kidney disease (ESKD), and the majority of those patients receive dialysis [1]. These patients have a comparable mortality and illness burden to cancer patients [2]. *Illness burden* is the impact of a patient’s symptoms and functional impairments on their life [3]. Common symptoms reported by patients associated with illness burden include pain, fatigue, nausea, interrupted sleep, and limited mobility [2, 4–6]. In addition, dialysis carries a significant *treatment burden*, defined as the objective *workload* (e.g. attending appointments) that patients must do to care for their health and the subjective impact of this workload on patient wellbeing [7]. Poorly coordinated care, frequent travel to appointments, and the constant and complicated navigation of administrative barriers to ongoing care all contribute to the overall treatment burden experienced by these patients [8]. In fact, the overall burden of dialysis leads many patients to voluntarily discontinue dialysis and die [9–11].

The Cumulative Complexity Model (CuCoM) describes how overwhelming amounts of patient workload can lead to worsening patient outcomes. Specifically, the CuCoM illustrates that patients must have adequate *capacity* to take on this workload. Those that do not, experience “workload-capacity imbalance.” [12] Workload-capacity imbalance can lead to difficulties in accessing and using healthcare, as well as enacting self-care tasks at home. These difficulties have downstream consequences on patient outcomes. Difficulty in access, use, and self-care is well-documented in the dialysis population with 25% of patients skipping or shortening dialysis sessions and 50% of patients reporting non-adherence to prescribed medications [13, 14]. Diminished health outcomes due to workload-capacity imbalance are also documented amongst patients living with ESKD [8], including increased risk of hospitalization and death [13]. The CuCoM also illustrates that as health outcomes worsen, both treatment and illness burden also worsen as healthcare practitioners try additional treatments to correct outcomes while the patient’s symptoms continue [12]. Despite the consequences of workload-capacity imbalance for patients, healthcare professionals often fail to understand patients’ workload related to treatment or their capacity to cope with this workload when prescribing treatments [15, 16].

Past research illustrates that despite the high objective workload of dialysis compared to many other chronic conditions, ESKD patients surprisingly report illness and treatment burden comparable to patients living with other chronic diseases, but those reporting the highest burden have significant capacity limitations [17]. However, we do not yet have an in-depth understanding of how patient experiences or self-management practices differ between low-burden and high-burden ESKD patients. Understanding such nuances is the first step toward developing interventions that lessen burden and improve patients’ adherence to therapy, health outcomes, and overall quality of life.

Thus, we examined the patient and healthcare practices associated with higher and lower levels of illness and treatment burden using an explanatory sequential mixed methods design, in which survey data was first collected and used to inform the selection of patients for qualitative interviews and observations.

## Methods

### Participant eligibility and recruitment

Mayo Clinic IRB approved this research (#18–000292). Written consent was obtained from participants. Participants were recruited from an academic medical center in the Midwestern United States with its own not-for-profit dialysis facilities, including two in-center dialysis units that serve 170 total patients. We invited participation from English-speaking patients prescribed maintenance dialysis at the medical center who had no major barriers to consent (e.g. cognitive impairment). Patients receiving in-center hemodialysis or receiving regular follow-up care for home dialysis modalities (either hemodialysis or peritoneal dialysis) were eligible.

During the initial recruitment, patients were approached at the two in-center dialysis units and the center’s outpatient clinic where patients receiving home dialysis underwent monthly follow-up. We approached all eligible patients for voluntary participation in these locations between April 3–24, 2018. Patients who consented completed their survey in person and were told they would be contacted at a future date if selected for interviews. At the time of interview, some participants had received a transplant; we did not exclude these patients from interview or observation. No compensation was provided for the survey, but patients were compensated for their participation in interviews and observations.

### Quantitative data collection

#### Survey administration

The survey took patients 10 to 15 minutes to complete independently. Study staff were available for questions while participants completed the survey or read questions aloud to participants if preferred.

#### Measures

*Demographic characteristics*. We abstracted patients’ age, gender, and marital status through chart review. Patients were asked on the survey about their number of months on dialysis, if they were currently listed for transplant, and whether their dialysis start was planned or unplanned. The full survey is attached as S1 Survey.

*Illness Intrusiveness Scale (IIS)* [18, 19]. We used the IIS, which was originally developed with dialysis patients, and also used widely in testing the Chronic Disease Self-Management Program [20]. In the dialysis population, it has previously been shown to be both valid and reliable (α = 0.81–0.85; test-retest reliability = 0.79) [19]. The IIS is a good measure of the impact of workload-capacity imbalance, as described by the CuCoM, on 13 areas of patients’ lives. Patients are asked questions such as: “How much does your illness and/or its treatments interfere with…” Example areas include: “your feeling of being healthy,” or “your relationship with your spouse or domestic partner.” [21] The extent to which treatment and illness impact each area are scored from 1 (not very much) to 7 (very much), resulting in scores ranging from 13 to 91. Items can also be marked as not applicable; these responses were coded as “1.” The overall IIS score was calculated as the sum of all individual item scores. The IIS subscales of were calculated as the means of the individual scores that went into each subscale: physical wellbeing and diet; work and finances; marital, sexual, and family relations; recreation and social relations; and other aspects of life.

#### Missing data

We made every effort to minimize missing data. Staff collecting surveys reviewed them for any missing items and confirmed with participants that they wished to skip any missing questions. Where any individual scores were missing, the overall score was also set to missing. Where any individual scores were missing, the subscales were also set to missing. Self-reported demographic information was treated as missing if participants chose not to answer a particular question.

### Quantitative data analysis

We first summarized demographics and IIS scores. Participants scoring in the highest IIS quartile were defined as having high burden, and participants scoring in the lowest IIS quartile were defined as having low burden. We then examined differences in demographic characteristics between high versus low burden groups using Fisher’s Exact Test for categorical variables and the Kruskal Wallis test for continuous variables. Statistical analysis was performed using SAS version 9.4 (Cary, NC) and p-values ≤0.05 were considered to be statistically significant.

### Qualitative participant selection

Following survey analysis, we purposefully invited all patients in the high (n = 16) and low (n = 17) burden groups, unless deceased, to return for an interview. In addition to these purposefully selected patients, four within the sample were recruited for ethnographic observations, described below. These were selected based on willingness to participate, IIS quartile, and richness of interview data.

### Qualitative data collection

Our research team (NE, SW, CML, AB) interviewed patients asking questions about patients’ day-to-day (dialysis and non-dialysis) schedule, activity management, and experiences with the healthcare system. The interview team was made up of two doctoral-level researchers and two bachelors-level researchers, all with training in qualitative interviewing and analysis. KRB oversaw interview training and progress. The interviews were conducted primary in the dialysis center, where patients have semi-private dialysis stations. Participants were also given the option to do the interview in in a private room at the healthcare center or in their home if they preferred. Semi-structured interviews were recorded and lasted 15 to 76 minutes (mean 40 minutes) [22]. All interviews were voice recorded, transcribed verbatim, and documented with field notes, which are notes taken by the qualitative researcher regarding observation/interview contextual details. The interview guide was iteratively revised throughout the first five interviews, and the final semi-structured interview guide is attached as S1 Interview guide.

Ethnographic observations were intended to supplement interview data by providing a view of patients’ day-to-day lives managing their ESKD. There is evidence that patients normalize many of the tasks that they do to manage their health [23], and therefore, these minutia may be omitted during experiential interviews but be visible during observation. KHP, KRB, and CML observed these participants’ practices in their daily living environments, including their in-center treatment location, homes, and workplaces. Participants were observed during two to four individual sessions, with up to eight hours of total time spent with each. Researchers took detailed ethnographic field notes, which were typed or voice-recorded and transcribed immediately after concluding each observation day.

### Qualitative data analysis

Our team analyzed 33 semi-structured patient interviews and 13 patient observation notes. Field notes from observations and interview transcripts were imported into Nvivo 12, a qualitative data management software. Data analysis generally followed an adapted Grounded Theory approach [24] without a set hypothesis a priori. We first began by reading observation notes and interviews. Five interviews were inductively coded by four coders (SW, NE, AT, PO) using line-by-line coding to create a codebook and calibrate usage of the codebook. The creation of the codebook was overseen by KRB, BT, AJP, and KP. After coders were calibrated, pairs of coders coded the remaining transcripts in duplicate. KRB then synthesized data using the Nvivo matrices feature. As patterns of coding clusters emerged, the query function was used to examine multi-code overlap. Finally, we summarized information into themes across patients as a whole, then looked more specifically at the high and low burden quartiles in comparison. All themes were derived inductively from the data.

During the process of synthesizing the data, KRB met with team members involved in the project to discuss the results and maintain trustworthiness of the interpretations. Throughout the process, the team responsible for analyzing the data, discussed each individual’s various perspectives and how these lenses influenced their perceptions of the data. Specifically, BT, AJP, and NE are clinicians, whereas SW, AT, PO, KP, and KRB were non-clinical researchers at the time of analysis. KRB is also a person living with chronic illnesses but does not live with CKD. During the analysis process, KRB also took detailed notes about her interpretations of the data and shared syntheses of those thoughts with members of the team during meetings to ensure additional perspectives were considered.

## Results

**Fig 1:**
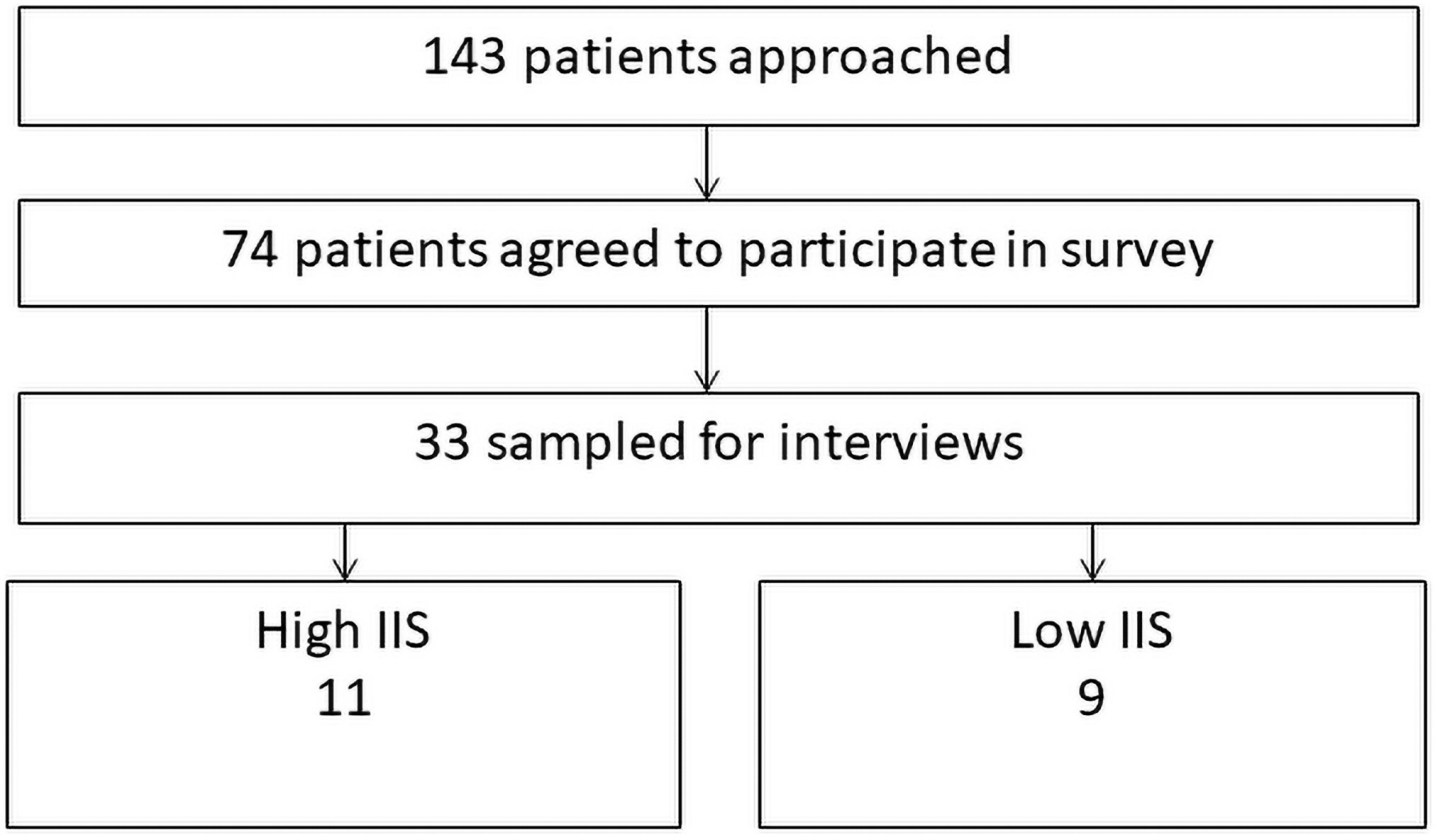
Participant recruitment.

Fig 1 demonstrates the participant recruitment results. Of 126 patients approached, 78 completed the survey for a total response rate of 61%. Of those invited for interview or observation, 33 agreed, 20 of whom were in the high or low burden groups for comparative analysis. Of the 20 patients in high or low burden groups interviewed, 3 patients in the highly burdened group were on a home dialysis modality and 1 in the low burden group. The remaining patients were on in-center hemodialysis. Given the small number of patients in the interview sample on home modalities available for comparison between high and low burden, we were not able to analyze these patients separately. Four patients completed observations.

### Quantitative findings

Patient characteristics are depicted in Table 1, including comparisons between high and low burden groups. Table 2 shows patient characteristics of the in-center versus home dialysis cohorts. The only statistically significant quantitative difference between the high and low burden groups was that participants experiencing high burden were significantly younger than those reporting low burden (48.4 vs 68.6, p = <0.001). In-center and home dialysis patients had no statistically significant differences except for sex.

**Table 1:** Patient demographic characteristics by patients reporting high versus low burden.

	Total (N = 78)	Low Burden* (N = 17)	High Burden* (N = 16)	P-value
**Age:**				0.0005^1^
N	78	17	16	
Mean (SD)	60.7 (17.27)	68.6 (16.81)	48.4 (11.36)	
Range	25.0, 87.0	29.0, 87.0	27.0, 65.0	
**Sex:**, n (%)				0.2960^2^
Male	50 (64.1%)	12 (70.6%)	8 (50.0%)	
**Race:**, n (%)				0.2803^2^
White	59 (75.6%)	16 (94.1%)	11 (68.8%)	
Black or African American	7 (9.0%)	1 (5.9%)	1 (6.3%)	
Asian	6 (7.7%)	0 (0.0%)	2 (12.5%)	
Other	4 (5.1%)	0 (0.0%)	1 (6.3%)	
Unavailable	2 (2.6%)	0 (0.0%)	1 (6.3%)	
**Ethnicity:**, n (%)				1.0000^2^
Hispanic or Latino	3 (3.8%)	0 (0.0%)	1 (6.3%)	
Not Hispanic or Latino	74 (94.9%)	16 (94.1%)	15 (93.8%)	
Choose Not to Disclose	1 (1.3%)	1 (5.9%)	0 (0.0%)	
**How long have you received dialysis (years)?**				0.6342^1^
N	73	16	15	
Mean (SD)	3.7 (3.78)	2.6 (1.64)	3.3 (2.79)	
**Was the decision to begin dialysis:**, n (%)				0.4905^2^
Planned	38 (52.8%)	11 (64.7%)	8 (50.0%)	
**Are you on a kidney transplant list?**, n (%)				0.1663^2^
Yes	24 (31.6%)	5 (29.4%)	9 (56.3%)	
**IIS score**				<.0001^1^
N	66	17	16	
Mean (SD)	43.0 (17.61)	22.8 (5.26)	67.3 (8.18)	
**Physical wellbeing, and diet subscale**				<.0001^1^
N	72	17	16	
Mean (SD)	4.5 (1.69)	3.0 (1.35)	6.0 (0.94)	
**Work and finances subscale**				<.0001^1^
N	72	17	16	
Mean (SD)	3.7 (2.10)	1.7 (0.90)	6.1 (1.04)	
**Marital, sexual, and family relations subscale**				<.0001^1^
N	68	17	16	
Mean (SD)	3.0 (1.69)	1.5 (0.64)	5.1 (0.80)	
**Recreation and social relations subscale**				<.0001^1^
N	71	17	16	
Mean (SD)	3.2 (1.40)	1.5 (0.58)	4.9 (0.78)	
**Other aspects of life subscale**				<.0001^1^
N	73	17	16	
Mean (SD)	2.8 (1.74)	1.4 (0.52)	4.4 (1.79)	

* Low Burden is defined as the lowest quartile of the Illness Intrusiveness Scale (core range 13–29); High Burden is defined as the highest quartile of the Illness Intrusiveness Scale (score range 58–81).

^1^Kruskal-Wallis p-value

^2^Fisher Exact p-value

**Table 2:** Patient demographics by in-center versus home dialysis.

	Total (N = 78)	In-center Dialysis (N = 63)	Home Dialysis (N = 15)	P-value
**Age:**				0.2115^1^
N	78	63	15	
Mean (SD)	60.7 (17.27)	61.8 (17.33)	55.8 (16.67)	
Range	25.0, 87.0	25.0, 87.0	29.0, 82.0	
**Sex:**, n (%)				0.0057^2^
Male	50 (64.1%)	45 (71.4%)	5 (33.3%)	
**Race:**, n (%)				0.4105^3^
White	59 (75.6%)	46 (73.0%)	13 (86.7%)	
Black or African American	7 (9.0%)	7 (11.1%)	0 (0.0%)	
Asian	6 (7.7%)	5 (7.9%)	1 (6.7%)	
Other	4 (5.1%)	4 (6.3%)	0 (0.0%)	
Unavailable	2 (2.6%)	1 (1.6%)	1 (6.7%)	
**Ethnicity:**, n (%)				0.5824^3^
Hispanic or Latino	3 (3.8%)	2 (3.2%)	1 (6.7%)	
Not Hispanic or Latino	74 (94.9%)	60 (95.2%)	14 (93.3%)	
Choose Not to Disclose	1 (1.3%)	1 (1.6%)	0 (0.0%)	
**How long have you received dialysis (years)?**				0.7310^1^
N	73	59	14	
Mean (SD)	3.7 (3.78)	3.8 (4.04)	3.0 (2.33)	
**Was the decision to begin dialysis:**, n (%)				0.5289^2^
Planned	38 (52.8%)	29 (50.9%)	9 (60.0%)	
**Are you on a kidney transplant list?**, n (%)				0.1218^2^
Yes	24 (31.6%)	16 (26.2%)	8 (53.3%)	
**IIS score**				0.3234^1^
N	66	51	15	
Mean (SD)	43.0 (17.61)	41.7 (16.80)	47.1 (20.17)	
**Physical wellbeing, and diet subscale**				0.8503^1^
N	72	57	15	
Mean (SD)	4.5 (1.69)	4.5 (1.70)	4.5 (1.70)	
**Work and finances subscale**				0.1647^1^
N	72	57	15	
Mean (SD)	3.7 (2.10)	3.5 (2.05)	4.4 (2.22)	
**Marital, sexual, and family relations subscale**				0.4894^1^
N	68	53	15	
Mean (SD)	3.0 (1.69)	2.9 (1.60)	3.3 (2.00)	
**Recreation and social relations subscale**				0.3731^1^
N	71	56	15	
Mean (SD)	3.2 (1.40)	3.2 (1.34)	3.5 (1.62)	
**Other aspects of life subscale**				0.4621^1^
N	73	58	15	
Mean (SD)	2.8 (1.74)	2.7 (1.73)	3.0 (1.80)	

^1^Kruskal-Wallis p-value

^2^Chi-Square p-value

^3^Fisher Exact p-value.

### Qualitative findings

We found travel as universally problematic amongst both patient groups. Additionally, we found five themes for which the illness experience of ‘high burden’ patients was significantly different than those reporting low burden. Demonstrative quotes from the themes that emerged from the comparative analysis are illustrated in Table 3.

**Table 3:** Patient quotes from high- versus low-burden groups.

*Theme*	*High Burden Example*	*Low Burden Example*
**Rhythm**		
• **Example: Energy management**	Well, I get up and eat breakfast, and then get ready for dialysis and go. I’ve gotten the pattern down. I wear a cutoff shirt, so my catheter’s exposed. It’s easier to get to. I get ready for dialysis. Then I come here for four hours. Then usually it makes me tired, so when I get home I sleep for about two hours covered up, cuz I’m cold. Then I get up and have supper and watch TV… it takes up all the day. • **[52 year old male, in-center dialysis]** I happened to have been the first patient at [center] I think to start nocturnal dialysis at home. I started that about three and a half months ago, and we’ve been working out little problems here, and there. My main problem that remains is that I just don’t sleep well during the either six or eight hours that I dialyze. I can sleep maybe two hours at the most. Sometimes I sleep almost none, and I just lay awake during those hours. As you can imagine, that’s quite stressful, and causes me not to feel good the next day. I feel really terrible usually the next day, but I have two days in between my dialysis runs at home. Then the second day, after I’ve gotten a good night of sleep in my own bed and everything, then I feel good. Nocturnal dialysis offers to hold many beneficial things for the patient that I’m really striving hard to work out the sleep issue. Right now that’s an issue, but I just met with my provider right before this appointment, and we’re going to try something for the sleep. I’m going to hope that that works, because I’m really driven to get to sleep better on those nights. If I slept better, I think so many things would. Yeah. • **[65 year old female, home dialysis]** I believe my time was 12:15, so noonish. I’m a late sleeper cuz I work overnights. I would stay up late. I would sleep until about time to go, probably about 11:30. I didn’t eat or drink cuz that would throw your weight off. I would go there and while I was there I chatted a lot, talked a lot. I really didn’t sleep. It’s hard to sleep there. Afterwards it’d be a four hour session and you’d be there about five hours. I would come home around 5:00 or so and go straight to bed. I would sleep about 12 hours. I mean it was long. It did make you really hungry though so I guess, yeah, I’m sorry I would eat when I got home and it’d be quite a bit. It makes you very hungry. Eat dinner and then I would just go straight to bed. Not much, it was not a productive day at all. • **[27 year old female, in-center dialysis]**	[On dialysis days] well, my husband wakes me up, because he gets up before me. He wakes me up about 5:10[AM]. It’s rush, rush, rush to get dressed, eat breakfast, and get down here by 7:30[AM]. Then if I have a fairly decent day here, if I don’t have cramps, and I don’t—my blood pressure doesn’t drop, I go home, and I eat lunch, and I usually sit down in the chair and probably take a little nap. After that I can get up, and I can do stuff, and work around the house. Now, today we’re gonna go away for a little overnight, because that’s about as long as we can be gone. Normally, I would go home and rest for a while, and then get up and iron [laughter], this type of stuff. [On non-dialysis days] It depends on the day of the week. I clean my house, or I should say my husband and I clean. We work together. We also work together getting meals. The other thing is I garden. On my non-dialysis days, I do exercises when I first get up in the morning. I sew. I like to read. I do lots of stuff. • **[75 year old female, in-center dialysis]** I, dialysis starts in the morning. They have me scheduled for 7:30 but I’m always here earlier because, like today, the reason I’m here earlier is because I can park out here for four hours. If there’s a meter that says if you have a wheelchair thing you can park there for four hours. I don’t have a wheelchair but my wife does. I use here so I get to park at where, and then if I’m done by 11:00 or so, then I’m hungry so I go home and, either that or stop and have something to eat. My wife is there, you know, she goes up to the cabin with my daughter and stuff. Otherwise, sometimes I just stop and have something to eat and then I go home. Sometimes, my son is a carpenter, and I will help him. I can’t really do much because my legs don’t, I can’t do certain things. I have a large yard and I sit in the tractor mower and I mow. I takes me about two hours to mow my yard and stuff. It’s something to do, you know… No, no, the dialysis does not bother me. I can, I’m, sometimes I will, when I come home, I may take a nap but most of the times I don’t. I just, I have things to do and my wife, she’s kind of, I have to buy all the groceries and stuff. She doesn’t, well, I do the cooking too. Anyway, so I always buy stuff I like. • **[87 year old male, in-center dialysis]**
• **Example: Dietary Restrictions**	Interviewee: Yeah. That was one of the things I hated the most was dealing with the nutritionist, because the doctor’ll say, “You need to watch how much phosphorous you eat. You gotta limit your salt intake,” or whatever. You’ll see the nutritionist. Then she basically hands you a pamphlet. These are the foods you should avoid. These are the foods you should have. I had one pamphlet. This is 100 mg of phosphorous. This is 200. This is 500. You should avoid this. Well, I don’t know how to put that into a meal plan. You ain’t helpin’ me at all. • **[48 year old male, home dialysis]** I already knew about the fluid restrictions and that was difficult in the beginning. With kidney disease, like I said I’ve had it my whole life so I’ve always drank a lot every day. It was difficult [changing to restricted fluids]. That was hard to take in the beginning and I shared with them that that would be difficult. They expressed how important it was and how it affects you physically. • **[27 year old female, in-center dialysis]**	The basics—lower potassium, lower phosphates, low sodium. I’m aware of what I’m eating versus just eating whatever I can. I’m just paying attention to what I’m eating and reading the nutritional facts. They told me only so much of this, or you should eat—‘cause I’m on the peritoneal, so it takes a lot more protein off—eat more protein. • **[29 year old male, home dialysis]** After I was diagnosed, after that initial diagnosis, I started—they told me about the diet, the renal diet: low sodium, low potassium, low fluid. We just basically went home, and we started just—we basically cleaned out the pantry, and we donated it to a neighbor. Then we went and we—they gave us all of our education for the diet, and then we went to the store, and we just refilled the pantry with renal-friendly foods. Yeah. Then we started making everything fresh at home, so we could control everything that was in the foods. We got nothing pre-made. We just made the food. • **[54 year old female, in-center dialysis]**
• **Example: Employment/ Hobbies**	There’s a lot of physical things that you can’t do anymore ’cause you don’t have the energy to do it or the stamina to do it. When your income goes from a working income to a disability income, that affects everything all the way down the line too. I just make the best of it, I guess. • **[55 year old male, in-center dialysis]** No, [work] would be difficult with the time. I couldn’t really do it after dialysis cuz I’m tired. I guess usually people are three days of dialysis, but I’m on four. • **[52 year old male, in-center dialysis]**	Not having a lot of—having a limited amount of time to actually see people and do things because you gotta be—I would imagine, too, for some people, not me personally, but balancing work and dialysis would be hard because some employers just don’t understand how important it is. I’ve heard horror stories of bosses who really don’t know that it’s a life and death situation, and they make people work, but for me personally, my employers always worked very well with whatever I had. Any type of appointment or if I had treatment, we made it so it was part of my availability. All those days were set, so I had them off for it, and then whenever—if an appointment suddenly jumped up the day before, they were very good about working around my appointment schedule. I was also very good about doing equal amounts for them, too. They worked with me because I worked with them. For example, on Black Friday, it’s our busiest of the year obviously because we’re retail. Instead of just saying, "Well, I can’t work Black Friday," ’cause my dialysis shift would go from Tuesday, Thursday, Saturday because Thursday’s a—Thanksgiving’s always on Thursday. We weren’t open. The dialysis center wasn’t open on Thursday, so people could have off with their families, so my dialysis schedule moved to a Tuesday, Friday, and Sunday that week. I would end up working on Black Friday and doing dialysis on Black Friday. Instead of saying, "Well, I can’t work on Black Friday. I have dialysis," I would be like, "Well, I can work from 5:00 a.m. to 11:00." If I was more willing to work for them, they would work for me sort-of-thing. • **[54 year old female, in-center dialysis]**
**Biographical Disruption**	[Emotionally I feel] good and not so good. I feel good that I’m managing my health, but on the other hand, you just feel like there’s so many things that you cannot do that you used to do, and it’s just sad. When I see people going to work and they’re making all this money, it hurts me a lot. That I can’t go to work anymore or I can’t manage to work a eight-hour shift anymore, to make that extra money for my kids. All I get a month is only 1,000. That barely pays for rent and other expense. We’re a little bit short on hand regarding that, so it makes me stress in a way. Emotionally, thinking about that. If I don’t think about that, and I just think about how I need to get better for my kids and all that, then I feel pretty good. • **[31 year old female, home dialysis]** Depressed ’cause I knew things were gonna change dramatically, and they did. Interviewer: Which are those things that you knew that would change? Interviewee: Me enjoying life and being able to do things that I enjoy. Everything gets taken away. Camping, swimming, hiking, outdoor activities, biking, four-wheeling, boating, traveling, going on vacation. All of it. • **[55 year old male, in-center dialysis]**	[I feel] good, actually, for the most part. With the exception of being attached to a machine three times a week, it was good. I was a runner. Well, I still am a runner. I would run between five and six miles, three times a week. Yeah. I followed my diet, and I exercised, kept active. I worked, and then—I worked Monday, Wednesday, and Friday, and every other weekend. Then Tuesday, Thursday, Saturday, I would dialyze. Then on the weekends that I worked, I would do a 5:00 a.m. to 11:00 shift, and I’d dialyze from noon to 4:00. It didn’t really affect me. I got married while I was on dialysis. I went on a honeymoon and traipsed around New York City while I was on dialysis. • **[31 year old male, in-center dialysis]** I don’t think it’s hindering too much. Interviewer: Mm-hmm. Maybe the times I have to do it. Say friends want to go out to the movies at 9:00 at night. I need to be getting my machine set up at that time otherwise—because I have to be hooked up to it for eight hours. Now, the movie is, say, half an hour away. Drive there half an hour. It’s a two-hour movie. Then, half an hour to drive home. What are we gonna do after? Now, my day tomorrow will be starting later and later and later. That would hinder it. … If you ask me how would it hinder me, I probably wouldn’t be able to answer that just because I wouldn’t know. • **[29 year old male, home dialysis]**
**Appraisal-Focused Coping Strategies**	At that time, I was still doing about everything. I was fishing and hunting and camping and riding my motorcycle. All that stuff you could still do by yourself I was still doing. Then, physically, it just got to where I couldn’t. I couldn’t ride the motorcycle anymore. I couldn’t ride the four-wheeler. I just wasn’t strong enough to ride safely anymore. I had to get rid of my camper because I wasn’t, obviously, taking my camper anyplace ’cause I had to do dialysis every other day. I got rid of that. Like I say, it’s been a slow process, but I watched everything just slowly get taken away. Everything. Everything except me and my life. I’m still here breathing. I’m not ready to go yet. Why? I have no idea, but I’m not ready yet. • **[55 year old male, in-center dialysis]** [On what they’d tell another dialysis patient just starting] That it’s gonna take up most of your life. Your whole life’s gonna be based around dialysis, no matter what. It’s gonna take all day. Even though it’s four hours, even if you do it in the morning, you’re still gonna need a nap. You’re gonna get cold and be weak after. Your life’s gonna change. • **[52 year old male, in-center dialysis]**	I haven’t had any real problems. A year ago I did have a—my graft got infected, so I was in the hospital for a while, but basically I’ve just worked my life around them. I have hypertension. I have hypothyroidism. I’ve got—[a lot going on]. I’m 75 years old, or 76 now, I guess. • **[75 year old female, in-center dialysis]** I didn’t know what it was all about until I guess I knew what it was for and stuff, but I didn’t know—everything that was involved with this. It’s all right. It’s a thing that’s gonna keep me alive until—it’s somethin’ that I have to do. Sometimes I think it’s almost like a job ’cause I’m here so much. • **[63 year old male, in-center dialysis]**
**Social Network**	All my friends. All of ’em. As soon as I got sick and had to quit drinking and wasn’t hanging out in the bars and wasn’t doing physical things anymore, all of ’em, they went their direction and I went my direction. I don’t see anybody anymore at all, which is too bad. That’s the way it worked out, but what do you do when you’re no fun anymore? You don’t do anything fun. You’re not fun. We’re going to where we can have fun. Okay. I can’t blame ’em. I might be the same way if I was in their situation. • **[55 year old male, in-center dialysis]** Right. Yeah. Right. Well I’ll tell you what it did to me personally way back when. Is that I worked at the same place for 25 years, and after they found out I needed my fourth transplant, they decided they didn’t need me anymore. I lost my job after 25 years. During the period of time that I had the disease, and dialysis for part of the time, and transplants, I became really, really—I isolated myself. I did not want to share my medical problems with the people I worked with. I did not really socialize with people at work very much, because I couldn’t just go out for a drink after work or whatever, like they might. I didn’t want to always be saying no, so I just pulled myself back from having a—I still have one very close friend from that period of time when I was working there. I really don’t have other friends who I stayed in contact with from there, which you’d think after 25 years you got a lot of them. I’ve always been a private person. I’ve always been an introvert. Okay. I like to socialize. I’m not saying that I don’t, but because I didn’t want to share my medical problems with people I’ve worked with for fear of how it would affect my career, I didn’t. That had became very isolating that way for work. • **[65 year old female, home dialysis]**	Oh, all of my children are always concerned what’s happening to dad. I see a lot of my—we have a good, close family relationship, very close, yeah. I usually will see some of my children every week. Yet I have one that lives half a mile from me, one daughter and her husband… We talk a lot about things that are happening, and that’s good. Then my two boys are involved with a business that I started, and that’s a big business. …. I’ve got one daughter, my youngest daughter who I was with yesterday, ‘cause it was her birthday, in [town]. Anyway, we see a lot of each other, my children. [wife]’s children, too. [Wife]’s children live closer by than mine, than the three. Again, known ‘em a lifetime, so it’s just like all family. • **[79 year old male, in-center dialysis]** [We] ust get up and have breakfast and watch TV. We go to town quite a bit and get groceries. We usually do that on a Friday or a Saturday. [My husband and I] do everything together, except when he’s on—he is on the road [for work]. … He is gone a couple times a week or something like that. He’s gone early in the morning, then he gets back around 5:00[PM] or 6:00[PM]. [When he is gone] I’m all by myself, but my son lives just next door. • **[78 year old female, in-cente dialysis]**
**Early Dialysis Journey Experiences**	Well, I start coming down here after about the first year of getting treatment in [city]. They had completely misdiagnosed my disease, and put me on really high dose steroids thinking they thought it was something else, and that that would cure it. High dose steroids are really hard on you. You blow up like this, and you retain fluid like crazy. Up there they never told me while on prednisone don’t eat salt. Salt will exacerbate the problem. I knew nothing about diet then. I didn’t know there were proteins, carbs, and fats. I had no idea of any of that. After about a year of that, I called down here myself, and made an appointment. Got an appointment in a couple of weeks—I think it was back then, and started seeing a nephrologist here who just amazing. In every nurse I saw, amazing, the treatment I got down here. I just continued to come here for all my medical needs. I’ve never gone anywhere else back up there, except when I had to for dialysis for a short time in center. • **[65 year old female, home dialysis]** I go, "I need to come in for a checkup. Because I don’t know why I’m—’cause I’ve been having a lot of those episodes. Where my tummy will hurt so bad, where I can’t walk." Then, so I went in, and she told me, oh, there’s really nothing that she could do for me. Because it’s just ovarian cysts and eventually it will just go away. I just have to take Tylenol. Then, she looked at my lab result from the ER, and she asked me, "Do you know that your kidney is abnormal?" I go, "No. Nobody told me anything. When I was in the ER, they didn’t tell me anything." Right then, she called my primary doctor. … She just called directly to my primary doctor and she was able to send me right over to him. He’s like, "Don’t panic. It’s probably because you’re sick. That’s probably why. Let’s do more tests and see where you’re at." After I did those tests, they told me, "Oh, yeah, you do have chronic kidney disease." He referred me to a specialist. At that time I started seeing that specialist. I just feel they weren’t taking good care of me. • **[31 year old female, home dialysis]**	I’d been seeing a kidney doctor before that and my creatinine levels kept going up and up. Before that I had a fistula started. I had the surgery for a fistula, and the doctor said eventually I probably would have to have dialysis, so I was prepared for it. • **[68 year old male, in-center dialysis]** I guess I’m a regular customer here three days a week [laughter], and I guess it’s gone quite well. I knew that [doctor] was the top one to put my fistula in my arm. He did a super job. I’ve been very fortunate to have wonderful medical care. I’ve had a chance to take dialysis—oh, I guess I’ve gone to [other state], twice to a sister-in-law, took dialysis there. Then last year I spent 12 days in [other state], and I took dialysis up there. That went fine, too. • **[79 year old male, in-center dialysis]**

#### Travel

Regardless of whether participants were in the cohort reporting low burden or high, they noted the difficult-to-overcome challenges of traveling while on dialysis. Many reported feeling tethered to their home environments. Most participants resorted to small day trips or a single overnight stay. Even in situations where patients had the opportunity for more extended travel, therapy as a visiting patient at other dialysis facilities was often fraught with unfamiliarity.

#### Rhythm of dialysis

Dialysis, regardless of modality, forced participants to learn a new rhythm of life. When people are well, their daily rhythms are designed for activities such as employment, volunteer work, caregiving for family, hobbies, and social life. However, once participants became sick enough to require dialysis, they required burdensome treatment and developed side effects that shifted their daily rhythms. This shift was evident regardless of modality. Following dialysis, participants reported having to learn to manage their own body’s reaction treatment, including fatigue and nausea, and realigning their daily activities around preparing for and recovering from dialysis. For example, one participant reported often working a full shift at his job before going to dialysis. He then learned that after many hours on his feet he had difficulty tolerating dialysis, so he began sleeping or lying down for at least five hours after his shift, prior to dialysis, which substantially impacted his work schedule. Typically, participants found that they needed anywhere from an hour to several hours to recover from their dialysis treatment.

While all participants needed to establish new rhythms after beginning dialysis, participants in the high burden group took longer to establish this new rhythm or felt that they could not establish one. The quotes from these participants reflected increased feelings of tiredness, low energy, or exhaustion, evidence that they are unable to establish routines to manage their energy outputs in comparison to their low-burden counterparts.

#### Biographical disruption

Biographical disruption is the disruption of one’s sense of self and social roles [25]. Participants in the high burden group reported more biographical disruption than those in the low burden group. Specifically, participants’ quotes indicated the greatest issues were shifts in their social roles of parenting and caregiving, an inability to remain dependable people within their social networks and friendships, and the loss of breadwinning abilities for their households.

#### Appraisal-focused coping strategies

Participants reporting low burden told stories about their approaches to life which were very different than the approaches of those reporting high burden. Specifically, people in the low burden group approached their situation with a more positive mindset than participants in the high burden group. The way they expressed their attitude toward dialysis and the language they used to describe their situation were positively framed (e.g. what they still could do despite needing dialysis vs. all the things they couldn’t do because of dialysis). Those reporting low burden also reported using more positive coping strategies (e.g. reading, learning, exercise).

#### Social network support

Participants reporting low burden described two elements of social support that differed from the high-burden participants. Low-burden participants reported that they had tight-knit social circles and perceived that their social networks continued to include and support them throughout treatment. In contrast, high-burden participants reported difficult social situations. For example, multiple patients in the high-burden cohort noted that they had once been social people. When they became sick and required dialysis, they were no longer able to inhabit their social spaces because of treatment restrictions and fatigue. Their friends did not make an effort to visit them at home or otherwise.

#### Early dialysis journey experiences

Finally, we noticed that participants in the high burden group discussed past experiences during the early part of their dialysis journeys differently than those in the low-burden group, irrespective of whether or not dialysis start was planned. In the high-burden group stories showed increased evidence of a lack shared decision making, in the choice to begin dialysis and/or the modality of dialysis, as well as more negative healthcare experiences early in their journeys. No participant reported current negative experiences with care.

## Discussion

### Summary of findings

This study utilized a mixed-methods design to gain an in-depth understanding of ESKD patients on dialysis reporting high versus low treatment and illness burden. Patients reporting high burden had more difficulty establishing a new rhythm of life on dialysis, more disrupted biographies, fewer appraisal-focused coping strategies, less supportive social networks, and more negative past healthcare interactions, which may have impacted how they experienced healthcare in the present. Quantitatively, we noted patients with high treatment and illness burden were significantly younger. While this initially may seem paradoxical, since chronic conditions accumulate with age, younger patients may be in different phases of individual and family development compared to their older peers. Younger patients may face greater personal or familial pressures that compete with their attention to healthcare such as paid employment and caregiving activities. This finding aligns well with the qualitative differences reported in the theme “biographical disruption,” attributed to the term first coined in Bury’s 1982. In his exploration of chronic illness as a disruptive event, he noted that the emergence of chronic illness disrupted both the perception of the self and the self in relationship with others [25]. Further, in studying the concept of hope in older patients living with chronic illness, it has been noted that older adults may reach for hope in different ways than younger individuals, namely through the processes of transcendence and positive reappraisal [26]. This is consistent not only with our finding that older patients report less burden, but also that those reporting reduced burden used positive reframing related to their situation. Our finding is also consistent with existing treatment burden literature, which has shown a greater percentage of patients under 60 years of age report unacceptable levels of treatment burden compared to those over 60 (51% versus 22%) [27].

We also found a significant difference in the number of males and females receiving in-center versus home hemodialysis or peritoneal dialysis. In examining the US Renal Data System 2018 incident cases, approximately 7 females begin any type dialysis for every 10 males [28]. Similar numbers persist across modality type, whereas in our sample, the ratios of females to males currently participating in-center dialysis versus a home modality was 2:5 versus 2:1. Therefore, we attribute this finding to sampling bias in our small sample.

### Comparison to existing literature

While we conducted our analyses inductively, the themes which emerged fit well with existing theory. The Theory of Patient Capacity (TPC) expands on the concept of capacity named in the CuCoM and states that patient capacity results from a dynamic interaction between patients and their **B**iographies, **R**esources, **E**nvironments, patient **W**ork, and **S**ocial Networks. The theme of “rhythm” fits well with the “patient work” construct, which proposes that when patients are given too many tasks at once, their capacity to act may be overwhelmed [29]. Furthermore, this theme suggests patients are tapping into their own personal “resource” of self-efficacy. The theme of “biographical disruption” overlaps with the construct of “biography,” which describes patients’ ability to overcome the disruptions in social roles and self-perception. The theme of “social network support” is in alignment with the “social network” construct, which suggests that patient capacity for self-care is influenced by robust, supportive social networks. Finally, the theme of “coloring experiences” is in alignment with the TPC’s “environment” construct, focused on patients’ interactions with their healthcare environments as principally responsible for their capacity to enact self-care.

Our study highlights some themes that are similar to others’ work in this area. For example, in a recent systematic review of 260 papers by Roberti et al. regarding the work associated with living on dialysis, it was regularly noted amongst studies that patients felt a lack of self-control around where and when they could travel [8]. Additionally, this review noted similar sources of social support, as well as the loss of social support that sometimes accompanied life on dialysis [8]. However, our work adds important information not previously highlighted. For example, while the review by Roberti et al. describes the work that is undertaken by patients on dialysis and the time management strategies required to organize that work [8], it does not discuss that the mastery of those task can be associated with less life disruption and a more positive treatment experience. The concept of biographical disruption in illness is not new, and has been found in patient narratives of those living with ESKD choosing to forego dialysis [30, 31]. However, our study appears to be the first to highlight this concept specifically amongst patients on dialysis.

Most importantly this study’s contribution to the literature is that the experience of dialysis is nuanced for each patient and burden is not universal. Some patients may feel a great deal of burden placed on their lives by dialysis whereas others experience less burden, and these two patient groups are different in their management of the disease and treatment. These differences may be amenable to interventions. For example, healthcare culture change and shared decision-making interventions may improve patients’ early dialysis journeys. Social support interventions may bolster patient networks where they are lacking, and capacity coaching [32, 33] interventions may improve patients’ abilities to establish a rhythm of dialysis and more positive framing of life with dialysis.

### Limitations and strengths

We first must acknowledge the limitations of this work. Principally, this research was conducted in a single healthcare system of an academic medical center within the upper Midwest of the U.S, limiting our ability to see themes that would arise in settings where structural inequity is more pronounced, as described by Roberti et al. [8]. We did not note these inequities, with the exception of occasional insurance complications and confusion. Furthermore, we had a slightly lower response rate than our previous survey only study (61% versus 70%). We suspect that this is due to the fact that participants at the time of consent were asked to participate in both the survey and the interview, if selected. Although they consented at the time of survey completion, when contacted for interviews, not all patients who were selected chose to participate in that portion of the study. Those who declined may have been feeling overly unwell, tired, or dealing with competing priorities. However, it is worth noting the demographic characteristics of our responders appears to be well representative of our population based on previous studies [17, 34, 35] and that response rates between 37% and 56% have been established under similar data collection conditions [36].

Despite these limitations, the strengths of this research are in its mixed-methods design and its ability to present nuances between patients reporting high versus low treatment and illness burden. Whereas other work has looked quantitatively or qualitatively at these issues in independence, our design allowed us to truly understand the differences between these patient populations, and therefore hypothesize interventions to improve the experience of patients reporting high burden based on findings from their low-burden counterparts. Furthermore, while our themes were inductively derived, they fit well with existing theory from a larger body of work across chronic condition populations. This suggests findings are likely transferable to other dialysis populations and reproducible in other illness populations as well.

### Practice and research implications

Hospitalization rates, emergency room utilization and readmission rates among dialysis patients are several standard deviations above the general United States Medicare cohort and strongly affected by patient adherence [37–40]. Decreasing these rates is of crucial importance directly to our dialysis-requiring population to improve quality of life and value. Currently, it is not standard practice in caring for patients on dialysis to measure their level of treatment and illness burden and respond accordingly. Furthermore, it is not current practice to adapt clinical actions based upon patients’ level of reported burden. However, this study highlights that this actually may be helpful to impact clinical outcomes, especially for patients with noted non-adherence.

This work provides important information to care teams as they design strategies to decrease suboptimal outcomes and further emphasize the importance of engaging our patients in developing care that fits. This work provides the foundational “preclinical phase” or “theory” portion of the design process [41], which is now translatable to intervention development. The work presented here is deeply steeped in the patients’ experiences of living on dialysis and navigating it in their own lives. This is a fundamental component to our team’s design process, which is in alignment with others in noting that integrating patient expertise into intervention design can offer valuable perspectives [42]. Managing health is an on-going task that goes beyond the fraction of their lives patients spend in the medical office or dialysis unit. There are many “on your own” hours that could be filled with patient peer support or peer coaching [43]. Based upon our data, this center’s teams are now in the development phase for an intervention that combines peer support and capacity coaching to patients specifically reporting high burden [32, 33]. Replication of this study’s concepts and designs among other demographic, socioeconomic, and geographic populations is necessary.

## Conclusion

Patients living on dialysis report high levels of illness and treatment burden as well as difficulties coping with these burdens. Until now, qualitative differences between patients reporting high versus low burden had not been elicited. Using a mixed methods design, we uncovered several important differences between these groups, which highlight important targets for future interventions designed to improve patients’ ability to cope with dialysis, adherence, and health outcomes.

## Supporting information

S1 Survey Participant survey. (DOCX)

S1 Interview guide Participant semi-structured interview guide. (DOCX)
