# Supplementary material for: Do patients with high versus low treatment and illness burden have different needs? A mixed-methods study of patients living on dialysis
Source: PLoS One. 2021 Dec 28;16(12):e0260914. doi: 10.1371/journal.pone.0260914 (PMC8714126; doi:10.1371/journal.pone.0260914)
Supplement: S1 Survey — (DOCX) [file pone.0260914.s001.docx]

**Today’s Date: _____ / _____ / __________**

**Thank you for helping with this study. Your answers are important to us. Please take the time to read and answer each question carefully by marking the box that best represents your answer. Some items ask you to fill in the blank space provided for your answer. Your responses are confidential. After completing this survey, please return it to the clinical staff that gave it to you. Please feel free to ask any questions you might have while completing the survey.**

1. **How would you describe your current health?**

_1_ Excellent

_2_ Very good

_3_ Good

_4_ Fair

_5_ Poor

**Please answer the following questions regarding your kidney disease and starting dialysis.**

1. **How long have you received dialysis? __________ months / years (circle one)**
2. **Was the decision to begin dialysis:**

_1_ Planned

_2_ Unplanned

1. **Did you begin dialysis:**

_1_ Inpatient / hospital setting

_2_ Outpatient / clinic setting

1. **Are you on a kidney transplant list?**

_1_ Yes

_2_ No

_3_ Choose not to answer

1. **Which of the following options were presented to you as treatment options to manage your renal failure? (Check all that apply.)**

_1_ Peritoneal dialysis

_2_ Home hemodialysis

_3_ Supportive care without dialysis

_4_ Renal transplant

_5_ None of the above

_6_ Choose not to answer

1. **Who mostly influenced your decision to start dialysis?**

_1_ Primary care provider

_2_ Nephrology provider

_3_ Family

_4_ Myself

_5_ Other, please specify: ________________________________________________________

_6_ Choose not to answer

1. **How do you currently feel about starting dialysis?**

_1_ Best decision I have made

_2_ Not as bad as I thought it would be

_3_ I thought it would be better, but I am okay with it

_4_ I wish I had never started dialysis

_5_ Other, please specify: ________________________________________________________

_6_ Choose not to answer

1. **I feel well prepared for what to expect with dialysis.**

_1_ Strongly agree

_2_ Agree

_3_ Undecided

_4_ Disagree

_5_ Strongly disagree

_6_ Choose not to answer

1. **How did your loved ones feel about your decision?**

_1_ Highly satisfied

_2_ Satisfied

_3_ Unhappy

_4_ Very unhappy

_5_ Uncertain

_6_ Choose not to answer

1. **The cost of dialysis played a role in my decision to start dialysis.**

_1_ Strongly agree

_2_ Agree

_3_ Undecided

_4_ Disagree

_5_ Strongly disagree

_6_ Choose not to answer

1. **Were the out-of-pocket costs of dialysis discussed prior to starting treatment?**

_1_ Yes

_2_ No

_3_ Choose not to answer

1. **Were the needs associated with dialysis (e.g., self-care, coming to appointments, etc.) discussed prior to starting treatment?**

_1_ Yes

_2_ No

**Please answer the following questions about prognosis (life expectancy).**

1. **Has anyone on your care team specifically discussed your prognosis (life expectancy) with you?**

_1_ Yes

_2_ No

_3_ Choose not to answer

1. **How well informed do you feel about your current prognosis (life expectancy)?**

_1_ Very well informed

_2_ Somewhat informed

_3_ Unsure

_4_ Somewhat uninformed

_5_ Completely uninformed

_6_ Choose not to answer

1. **How do you expect your health to be in 12 months from now?**

_1_ Much better

_2_ Somewhat better

_3_ The same

_4_ Worse

_5_ Much worse

_6_ Choose not to answer

1. **If you were seriously ill, would you prefer care to:**

_1_ Extend life, even if it meant more pain and discomfort

_2_ Relieve pain and discomfort, even if it meant not living as long

**Please fill out the following questions related to your kidney disease and the dialysis treatments you use to manage it.**

1. **How much do your illness and/or its treatment interfere with…**

**your feeling of being healthy?**

| _0_ | _1_ | _2_ | _3_ | _4_ | _5_ | _6_ | _7_ |
| --- | --- | --- | --- | --- | --- | --- | --- |
| Not applicable | Not very much |  |  |  |  |  | Very much |

**the things you eat and drink?**

| _0_ | _1_ | _2_ | _3_ | _4_ | _5_ | _6_ | _7_ |
| --- | --- | --- | --- | --- | --- | --- | --- |
| Not applicable | Not very much |  |  |  |  |  | Very much |

**your work, including job, house work, chores, or errands?**

| _0_ | _1_ | _2_ | _3_ | _4_ | _5_ | _6_ | _7_ |
| --- | --- | --- | --- | --- | --- | --- | --- |
| Not applicable | Not very much |  |  |  |  |  | Very much |

**playing sports, gardening, or other physical recreation or hobbies?**

| _0_ | _1_ | _2_ | _3_ | _4_ | _5_ | _6_ | _7_ |
| --- | --- | --- | --- | --- | --- | --- | --- |
| Not applicable | Not very much |  |  |  |  |  | Very much |

**quiet recreation or hobbies, such as reading, TV, music, knitting, etc.?**

| _0_ | _1_ | _2_ | _3_ | _4_ | _5_ | _6_ | _7_ |
| --- | --- | --- | --- | --- | --- | --- | --- |
| Not applicable | Not very much |  |  |  |  |  | Very much |

**your financial situation?**

| _0_ | _1_ | _2_ | _3_ | _4_ | _5_ | _6_ | _7_ |
| --- | --- | --- | --- | --- | --- | --- | --- |
| Not applicable | Not very much |  |  |  |  |  | Very much |

**your relationship with your spouse or domestic partner?**

| _0_ | _1_ | _2_ | _3_ | _4_ | _5_ | _6_ | _7_ |
| --- | --- | --- | --- | --- | --- | --- | --- |
| Not applicable | Not very much |  |  |  |  |  | Very much |

1. **How much do your illness and/or its treatment interfere with…**

**your sex life?**

| _0_ | _1_ | _2_ | _3_ | _4_ | _5_ | _6_ | _7_ |
| --- | --- | --- | --- | --- | --- | --- | --- |
| Not applicable | Not very much |  |  |  |  |  | Very much |

**your relationship and social activities with your family?**

| _0_ | _1_ | _2_ | _3_ | _4_ | _5_ | _6_ | _7_ |
| --- | --- | --- | --- | --- | --- | --- | --- |
| Not applicable | Not very much |  |  |  |  |  | Very much |

**social activities with your friends, neighbors, or groups?**

| _0_ | _1_ | _2_ | _3_ | _4_ | _5_ | _6_ | _7_ |
| --- | --- | --- | --- | --- | --- | --- | --- |
| Not applicable | Not very much |  |  |  |  |  | Very much |

**your religious or spiritual activities?**

| _0_ | _1_ | _2_ | _3_ | _4_ | _5_ | _6_ | _7_ |
| --- | --- | --- | --- | --- | --- | --- | --- |
| Not applicable | Not very much |  |  |  |  |  | Very much |

**your involvement in community or civic activities?**

| _0_ | _1_ | _2_ | _3_ | _4_ | _5_ | _6_ | _7_ |
| --- | --- | --- | --- | --- | --- | --- | --- |
| Not applicable | Not very much |  |  |  |  |  | Very much |

**your self-improvement or self-expression activities?**

| _0_ | _1_ | _2_ | _3_ | _4_ | _5_ | _6_ | _7_ |
| --- | --- | --- | --- | --- | --- | --- | --- |
| Not applicable | Not very much |  |  |  |  |  | Very much |

**Please think about the decision you made about starting dialysis to treat your kidney disease. Please show how you feel about these statements by selecting a response from Strongly Agree to Strongly Disagree.**

|  | **Strongly Agree** | **Agree** | **Neither Agree nor Disagree** | **Disagree** | **Strongly Disagree** |
| --- | --- | --- | --- | --- | --- |
| 1. **It was the right decision** | _1_ | _2_ | _3_ | _4_ | _5_ |
| 1. **I regret the choice that was made** | _1_ | _2_ | _3_ | _4_ | _5_ |
| 1. **I would go for the same choice if I had to do it over again** | _1_ | _2_ | _3_ | _4_ | _5_ |
| 1. **The choice did me a lot of harm** | _1_ | _2_ | _3_ | _4_ | _5_ |
| 1. **The decision was a wise one** | _1_ | _2_ | _3_ | _4_ | _5_ |
