# Supplementary material for: Do patients with high versus low treatment and illness burden have different needs? A mixed-methods study of patients living on dialysis
Source: PLoS One. 2021 Dec 28;16(12):e0260914. doi: 10.1371/journal.pone.0260914 (PMC8714126; doi:10.1371/journal.pone.0260914)
Supplement: S1 Interview guide — (DOCX) [file pone.0260914.s002.docx]

Participant #_______________

Date_______/_____/_______

Interview Guide

Hi, thank you for coming in today. My name is [researcher name]. I am a researcher here, at Mayo Clinic. We are doing this study because we want to learn more about what your life on dialysis is like.

This interview will take about 60 minutes. The interview will focus on your personal experience of managing kidney disease and dialysis treatment.

I will ask you a series of questions which follow a loose structure, and I will ask you follow up questions based on your answers. As an interviewer, my job is to learn about how you live with dialysis in a lot of detail, so I will ask questions that may at times seem boring or overly detailed but actually help us to understand your life and experiences in a lot of depth. We can veer away from the set of questions I have in front of me but I may at times bring us back to this set of questions to make sure I get all of the information I need. Also, please be aware that I will try to stay neutral during this process so it may feel a little more awkward than your average back and forth conversation. I may take notes while you talk, but even if I’m not making eye contact I am listening to you.

**Do I have your permission to voice record this interview so that later, I can go back and make sure I transcribe this conversation accurately?** Ok, I will put this recorded somewhere where it will pick up both of our voices. I’ll check it from time to time to make sure it is recording but we can mostly forget about it.

Everything you say in our interview is confidential. I am part of an independent research group at Mayo Clinic, and not a part of the clinical care team. You can feel free to share your honest opinions with me, and I will not directly share them with the people who provide your care. The only reason we would inform your clinical care team of anything would be if we believe you could be at risk of harming yourself or those you love.

Though quotes from our conversations may appear in research publications or be used in academic workshops or presentations, your name will not be linked to this information. We may change details that don’t matter very much to the meaning of your words in order to further keep your identity private. If at any time you feel uncomfortable answering a question and would like to skip it, would like to stop the use of the recorder or the interview itself, please let me know. I am very interested in your day-to-day experience.

The overarching reason for talking with you today is to understand what your experience on dialysis is like, what you do to manage your health while on dialysis, and your feelings about starting dialysis.

1. Tell me how things have been going in managing your health lately?
   1. *What other conditions are you managing in addition to your Kidney Disease?*

1. Would you mind just walking me through what the process looked like when you began dialysis from the moment someone mentioned to you that you might need or would be starting dialysis?
   1. Probes:
      1. *What was going on in your life during that time?*
      2. *What was going on with your health during that time?*
      3. *How was the potential need to go on dialysis brought up to you?*
      4. *Who was there supporting you during that time?*
      5. *What role did each person play during your decision making process?*
      6. *What were your feelings about starting dialysis before it began?*
2. I want to understand your feelings about dialysis now that you have been on it for some time.
   1. Can you tell me about how you feel now about dialysis, compared to before you began dialysis?
   2. Probes:
      1. *What makes you feel that way?*
      2. *What, if any, are the things you wish you would have known before beginning?*

I’d like to understand what your day-to-day activities of managing dialysis are. The next two questions will ask for you to give us a detailed account of what you do on dialysis and non-dialysis days.

1. What does your typical full day look like on your dialysis days? Start with what you do when you first wake up in the morning and go from there.
   - 1. PROMPT: What next?
     2. PROMPT: Can you give me more detail on that?
        1. *Activities to ask them about-- check items off as they come up in the interview ASK FOR DETAIL*
           1. *Grocery shopping* ☐
           2. *Planning meals* ☐
           3. *Food preparation/eating* ☐
           4. *Taking medication* ☐
           5. *Organizing/managing medication* ☐
           6. *scheduling* ☐

*related to medical needs* ☐

*related to other (work* ☐ *social* ☐ *family needs* ☐*)*

- - - - 1. *transportation to the clinic* ☐
        2. *setting up dialysis (for at-home)* ☐
        3. *coordinating with support person(s)* ☐
        4. *for participants who work: carrying out work activities* ☐
        5. *managing bodily symptoms (e.g. fatigue, cramps, nausea, limited freedom of movement, headache)* ☐
        6. *planning ahead for sitting during tx* ☐
        7. *passing time during tx (e.g. sleep, watch TV, read)* ☐
  1. How manageable are these specific activities are that are involved in your care?
     1. Probe
        1. *Are there certain activities that are easier?*
           1. *What makes you think/say that?*
        2. *Are there certain activities that you struggle with more than others?*
           1. *What makes you think/say that?*

1. What does your typical full day look like on your non-dialysis days? Start with what you do when you first wake up and so on
   - 1. PROMPTS: What next?
     2. PROMPTS: Can you tell me more about that?
        1. *Activities to ask them about-- check items off as they come up in the interview and ASK FOR DETAIL*
           1. *Grocery shopping* ☐
           2. *Planning meals* ☐
           3. *Food preparation/eating* ☐
           4. *Taking medication* ☐
           5. *Organizing/managing medication* ☐
           6. *scheduling* ☐

*related to medical needs* ☐

*related to other (work* ☐ *social* ☐ *family needs* ☐*)*

- - - - 1. *transportation to the clinic* ☐
        2. *setting up dialysis (for at-home)* ☐
        3. *coordinating with support person(s)* ☐
        4. *for participants who work: carrying out work activities* ☐
        5. *managing bodily symptoms (e.g. fatigue, cramps, nausea, limited freedom of movement, headache)* ☐

1. Who participates in daily activities with you?
   - 1. *Specific people they name – In what ways does that person support you? Note for each person whether they facilitate emotional/social support OR instrumental/practical support OR both* ☐
     2. *Are there people you used to spend more time with before you were on dialysis?* ☐
2. Tell me about how have your daily activities now have changed from your daily activities before you began dialysis?
   1. Probes
      1. *Working* ☐
      2. *Volunteering* ☐
      3. *Hobbies* ☐
      4. *Caregiving* ☐
      5. *Social activities* ☐
      6. *Others?* ☐
3. Tell me about the people who help you with your care at the clinic?
   1. Probes:
      1. *What specific activities that these individuals do that are helpful*
      2. *What do you find frustrating when interacting with your care or the health care environment in general?*

The next questions deal with feeling and perspectives of living on dialysis

******If patient is expressing they feel no joy in their life, locate most trusted Mayo clinician to reach out to inform them of depression/suicidal-related concern about said patient*******

1. Where do you find the most joy in your life?
   1. *Probes:*
      1. *In what ways has dialysis helped you maintain {this joyful activity}?*
      2. *In what ways has dialysis hindered you in keeping up with {this joyful activity}?*
   2. *Would your answer to this question have been different before you began dialysis?*
      1. *If yes, how so?*
      2. *e) If no, proceed to question 10*
2. What do you find are the biggest problems of being on dialysis?
   1. *How have these changed over time?*
   2. *Did these meet your expectations of the problematic pieces?*
3. What do you find are the least problematic parts of being on dialysis?
   1. *How have these changed over time?*
   2. *Did these meet your expectations of the least problematic pieces?*

The next questions are more specifically about the process of starting dialysis specifically, so I will ask you reflect about the past as well as the present.

1. What types of information were you given just before or at the time you began dialysis?
   1. *Information about cost, about frequency, about lifestyle requirements, etc.*
2. In what ways was that information helpful in beginning dialysis?
3. In what ways was that information not helpful, frustrating, or overwhelming in beginning dialysis?
4. Choices related to dialysis treatment could be: about when to begin dialysis, different types of dialysis, or to begin versus not begin dialysis. Did someone mention to you any of these types of choices during the time of starting dialysis?

- *If yes, which choices were mentioned? Can you tell me more about them?*
- *If no, thank patient for answering and proceed to question 16.*

1. Who was it that offered those choices and what did your conversation with that person look like?
   1. *What were your feelings about those choices at the time?*
   2. *What are your feelings about those choices now?*
2. Who helped you in making those choices?
   1. *Tell me more about that person’s role in the conversation or in your decisions related to dialysis treatment.*
3. What would you like other people potentially facing dialysis start to know?

****CLOSING QUESTION****

1. What else would you like share about your feelings about starting dialysis or being on dialysis?
